# Highly spatial imaging of electrochemical activity on the wrinkles of graphene using all-solid scanning electrochemical cell microscopy

**DOI:** 10.1016/j.fmre.2021.08.001

**Published:** 2021-08-19

**Authors:** Rong Jin, Hong-yan Lu, Lei Cheng, Jian Zhuang, Dechen Jiang, Hong-Yuan Chen

**Affiliations:** a State Key Laboratory of Analytical Chemistry for Life Science, School of Chemistry and Chemical Engineering, Nanjing University, Nanjing, Jiangsu, 210023, China; b School of Mechanical Engineering, Xi'an Jiaotong University, Xi'an, Shanxi, 710049, China

**Keywords:** Scanning electrochemical cell microscopy, Solid electrolyte, High spatial resolution, Electrochemical activity, Wrinkle

## Abstract

Here, all-solid scanning electrochemical cell microscopy (SECCM) is first established by filling polyacrylamide (PAM) into nanocapillaries as a solid electrolyte. A solid PAM nanoball at the tip of a nanocapillary contacts graphene and behaves as an electrochemical cell for simultaneously measuring the morphology and electrochemical activity. Compared with liquid droplet-based SECCM, this solid nanoball is stable and does not leave any electrolyte at the contact regions, which permits accurate and continuous scanning of the surface without any intervals. Accordingly, the resolutions in the lateral (*x*-*y*) and vertical (*z*) directions are improved to ∼10 nm. The complete scanning of the wrinkles on graphene records low currents at the two sidewalls of the wrinkles and a relatively high current at the center of the wrinkles. The heterogeneity in the electrochemical activity of the wrinkle illustrates different electron transfer features on surfaces with varied curvatures, which is hardly observed by the current electrochemical or optical methods. The successful establishment of this high spatial electrochemical microscopy overcomes the current challenges in investigating the electrochemical activity of materials at the nanoscale, which is significant for a better understanding of electron transfer in materials.

## 1 Introduction

Graphene has attracted extensive attention due to its excellent electrical and mechanical properties [1], [2], [3], [4]. Chemical vapor deposition (CVD) is the most popular method to prepare large and high-quality graphene [5]. For subsequent applications, graphene grown on the surface of metal often needs to be transferred to other substrates by dissolving the supporting metal. Recent studies have shown that graphene floating on the surface of a solution forms wrinkles due to the surface tension of water, which breaks the uniformity of electrons in graphene [6], [7], [8], [9]. Accordingly, electron transfer is inhibited, resulting in graphene-based devices with low performance [10], [11], [12], [13]. However, some other studies report that wrinkles with a fold structure increase the specific surface area and inhibit stacking, resulting in improved energy storage performance compared to flat graphene [14,15]. Thus, locally measuring the electron transfer rate of the wrinkles of graphene is the key to solving this controversy, which requires a highly spatial electrochemical mapping method.

Scanning electrochemical microscopy (SECM) is a scanning probe technique with high resolution that uses the faradic current as feedback to image the topography or local reactivity of the substrate [16], [17], [18]. A recent breakthrough has pushed this technique to map the electrocatalytic activity of semi-two-dimensional catalysts with single-edge sensitivity (15 nm spatial resolution in the x-y directions) [19]. Despite the tremendous progress in SECM imaging, this approach has difficulty comapping the electrochemical activity and morphology of the material based on faradic current. [20] In addition, the resolution in the z direction is relatively low, which hinders the study of a three-dimensional (3D) surface. Although some other techniques, such as scanning electron microscopy (SEM) and atomic force microscopy (AFM), could be incorporated to provide high-spatial structural information, some mismatch in the colocation using these microscopies and SECM is unavoidable. [21], [22], [23], [24] To address the costudy of the electrochemical activity and morphology at the surface, scanning electrochemical cell microscopy (SECCM), another highly spatial mapping approach, was developed [25], [26], [27]. Individual nanometer-sized liquid droplets are formed at the material surface as electrochemical cells. By recording the motor position where the nanodroplet is in contact with the surface and the faradic current in this droplet, the electrochemical activity and morphology at the surface were obtained simultaneously. However, maintaining the same size of liquid droplets during SECCM scanning, especially at 3D surfaces with different curvatures, is not easy, which affects the accurate characterization of the electrochemical activity [28]. The development of a more robust electrochemical imaging approach is still needed to study the electrochemical features at 3D surfaces, such as graphene with wrinkles.

**Fig 1:**
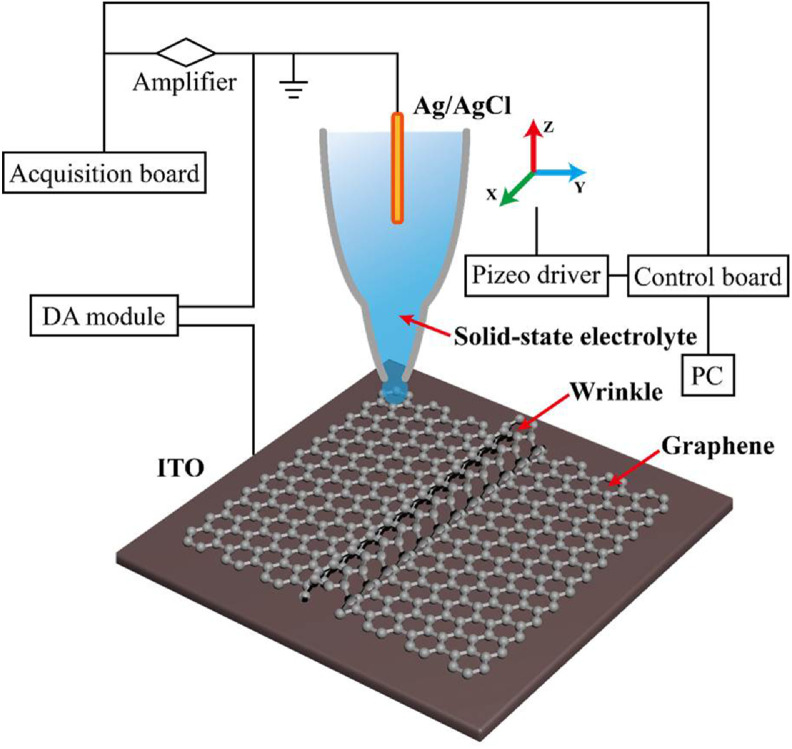
**All-solid SECCM for high spatial characterization of the electrochemical activity of wrinkled graphene**.

In this study, all-solid SECCM is established by utilizing polyacrylamide (PAM) as a solid electrolyte to replace the traditionally used liquid electrolyte. A solid PAM nanoball is formed at the tip of the nanocapillary that is in contact with graphene to provide information about the morphology and an initial electrochemical measurement, as illustrated in Fig. 1. Compared with the liquid droplet, the solid nanoball is stable and offers the same contact region at individual sites of graphene. Moreover, no droplet remains on the surface of the material after each contact using the solid nanoball, which avoids the inevitable droplet interval in the liquid SECCM mode. Eventually, this new all-solid SECCM should achieve continuous mapping of the surface with a high spatial resolution. The characterization of electrochemical activity at the graphene clearly illustrates an inhibited electron transfer at the wrinkles compared with flat graphene. More importantly, continuous mapping reveals that the electron transfer rate only decreases at the sidewall of the wrinkle with a small curvature, which is partially recovered at the center of the wrinkle. This new finding from highly spatial electrochemical mapping should offer more information to understand the effect of wrinkles on the properties of graphene.

## 2 Materials and methods

### 2.1 Chemicals and reagents

Borosilicate capillaries (BF100-58-10) and quartz capillaries (QF100-50-7.5) were obtained from Sutter Instrument Co. (Novato, CA). All chemicals were purchased from Sigma–Aldrich Co. (Saint Louis, USA), unless indicated otherwise. Ultrapure water with a resistivity of 18.2 MΩ/cm was used throughout. All chemicals and reagents were used as received without further purification.

### 2.2 Preparation of graphene sample

Multilayer graphene prepared by the CVD method was purchased from Xianfeng Nano Tech. Co. (Nanjing, China). The graphene on the supporting Cu foil was cut into 2 × 2 mm pieces, which were floated on the surface of a 1 M FeCl_3_ solution for 1 h. After the complete dissolution of the Cu foil, the floating graphene films were captured by indium tin oxide (ITO) slides. The ITO slide was half immersed in water and moved near the graphene. Since both the ITO surface and graphene are hydrophobic, graphene adsorbs on the ITO surface. Then, the ITO slide was removed from the water with the adsorbed graphene. The sample was washed with water and ethanol 3 times and dried with N_2_.

### 2.2 Preparation of solid electrolyte filled nanocapillary

The nanocapillaries (o.d. ∼20 nm) were prepared from quartz capillaries (o.d. 1.0 mm, i.d. 0.58 mm) using a P-2000 micropipette puller (Sutter Instrument, USA). The program was set as follows: Line 1: Heat 900 Fil 3 Vel 20 Del 125 Pul 120; Line 2: Heat 950 Fil 3 Vel 20 Del 125 Pul 180. The solid electrolyte in the nanocapillary was prepared by the photoinitiation of free radical polymerization. The monomer consisted of 50 wt% AM (acrylamide), 20 wt% HEA (hydroxyethyl acrylate), 30 wt% PEGDA (polyethylene glycol diacrylate) and 500 mM KCl. The ratio of water and monomer was 4:6, and the photoinitiator was 2-hydroxy-2-methyl-1-phenylacetone with a content of 1 vol%. The light source was 365 nm UV light with a power of 6 W. After filling with the monomer solution, the nanocapillary was pressed under a pressure of 0.2 MPa using an air pump so that a nanodroplet of monomers egressed from the tip of the nanocapillary. Then, polymerization was initiated under illumination for 5 min. The morphology of the nanocapillary was characterized by optical microscopy (Nikon Ti, Japan) and scanning electron microscopy (SEM, Hitachi S-4800, Japan). Before SEM characterization, the nanocapillary with the solid-state electrolyte inside was freeze-dried. While not in use, the nanocapillary was stored in the electrolyte with the same ion concentration to maintain the ion concentration and water content inside the nanoball.

### 2.3 SECCM imaging

The nanocapillary filled with solid electrolyte was installed on a piezomotor (P-753, PI, USA), and the sample was installed on another piezomotor (P-621, PI, USA). Ag/AgCl wire was inserted into the nanocapillary as a quasi-reference counter electrode. During the scanning process, the nanocapillary moved in the xyz direction. For each scanning point, the nanocapillary stopped moving for 100 ms after touching the sample to collect the electrochemical signal. The position of the nanocapillary was recorded to obtain information about the surface morphology. To decrease the current noise, a low noise amplifier (DDPCA-300, FEMTO, Germany) was used that had a standard noise of 0.4 fA and a maximum gain of 10^13^ V/A. In addition, the microscope, piezoelectric motor, amplifier and electrical machinery were positioned inside a Faraday cage to realize multilayer electromagnetic shielding. The controller, PC and other electrical equipment were placed as far away from the microscope as possible to avoid any electromagnetic interference. The control program was written in the C language. For the characterization of the SECCM resolution in the x-y direction, a solid electrolyte containing 500 mM KCl was filled into the nanocapillary that was used to contact an Au-coated ITO electrode. The Au film was prepared by an evaporator with a current of 15 mA and an evaporation time of 120 s. A bias of 2 V was applied at the Au-coated ITO electrode, which induced electrochemical corrosion of the Au layer at the contact region. The dimensions of the contact region were then characterized by SEM.

To map the morphology and electrochemical activity of graphene, a nanocapillary containing a solid electrolyte (500 mM KCl, 100 mM K_4_[Fe(CN)_6_] and 100 mM K_3_[Fe(CN)_6_]) was adapted. For each contact point, the current and position of the three axes of the piezoelectric motor were recorded synchronously. MATLAB was used to analyze the current and the xyz position of each scanning point at the contact moment. SPIP (6.0.10) was used to remove the noise, and a linewise horizontal trim was adopted to revise the substrate slope.

### 2.4 Calculation of the standard rate constant (k_0_)

According to the relationship between the current and potential characteristics, the current (*i*) was in direct proportion to k_0_:

(1) $$i=\text{FA}k_{0}\left[C_{O}\left(0,t\right)e^{-\alpha f\left(E-E^{\Theta }\right)}-C_{R}\left(0,t\right)e^{\left(1-\alpha \right)f\left(E-E^{\Theta }\right)}\right]$$

where F is the faradaic constant, A is the electrode area, C_O_(0,t) and C_R_(0,t) are the oxide and reduction concentrations with the distance away from the electrode surface (x) and time (t), α is the transfer coefficient, E is the applied potential and E^Θ^ is the standard electrode potential [29]. The parameter f in Eq. 1 is a constant that can be expressed as f=F/RT, where *F* is 96485 C/mol, *R* is 8.314 J/(mol•K), and *T* is 298.15 K. Thus, f is calculated to be 38.92 C/J. A is equal to the area of the circle whose diameter is 10 nm, C_O_(0,0) and C_R_(0,0) are both 100 mM, *α* is 0.5, E^Θ^ is 0.5833 V vs. Ag/AgCl and *E* is 0.5 V. Eq. 1 is only suitable for some special cases. The interface must be under balanced conditions, where the concentrations of the oxidative and reductive species are the same. In this work, the concentrations of K_4_[Fe(CN)_6_] and K_3_[Fe(CN)]_6_ were both 100 mM. Since the reaction lasted for only 100 ms, the concentrations of both species should be approximately invariable. Therefore, Eq. 1 could be applied in our study, and k_0_ was calculated to be 0.0263 cm/s when *i* was 1 pA. The measured currents at each site in the SECCM image were then converted into *k_0_* to better illustrate the electron transfer rate of the wrinkles on graphene.

## 3 Results and discussion

### 3.1 Characterization of the solid electrolyte nanoballs at the tip of the nanocapillary

The formation of highly conductive solid-electrolyte nanoballs at the tip of the nanocapillary is the key to achieving all-solid SECCM. The conductivity of the solid electrolyte with 500 mM KCl was measured and compared with that of aqueous solution containing 500 mM KCl. The current flowing through the solid electrolyte is approximately 40% of the current collected from the aqueous electrolyte (Fig. S1, SI), which is mainly ascribed to the restricted electron transfer in the crosslinked polymer. Despite the relatively low conductivity of solid electrolytes, they should still be qualified as electrolytes for electrochemical measurements.

**Fig 2:**
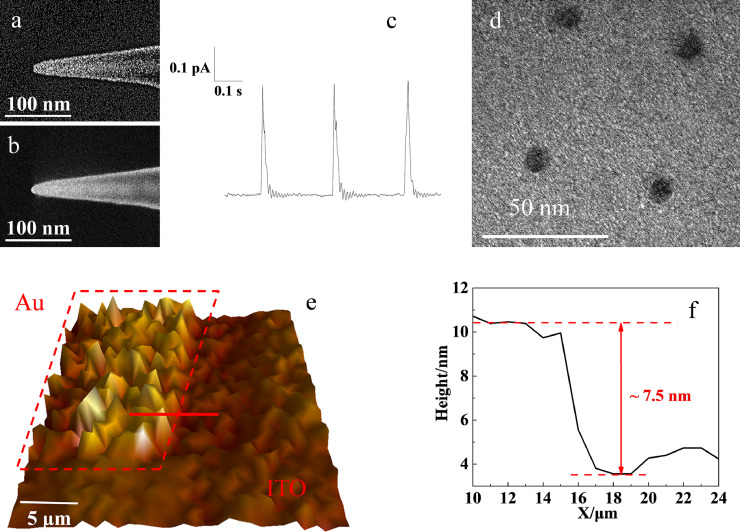
(a), (b) SEM image of the nanocapillary with an opening of 20 nm (a) and the capillary filled with solid electrolyte exhibiting a nanoball at the tip (b); (c) the typical current trace recorded during the consecutive contacts by the nanoball at ITO surface; (d) SEM image of the Au layer after the contact with the nanoball and the electrochemical corrosion; (e) the SECCM image of Au layer at the ITO surface using the nanocapillary filled with the solid electrolyte; and (f) the height difference between Au layer and ITOs surface as marked with red line in Figure 2.

Then, the monomers are filled into the nanocapillary to prepare solid electrolyte nanoballs at the tip. For a high spatial resolution, a nanocapillary with an outer diameter of 20 nm and inner diameter of 10 nm was used, as characterized by SEM images (Fig. 2a and Fig. S2a, SI). Simple polymerization in the capillary under illumination results in inevitable contraction that cannot form protrusions outside the capillary. To address this problem, a pressure of 0.2 MPa is applied inside the capillary during polymerization. After 5 min, a nanoball with a diameter of ∼10 nm is formed at the tip of the capillary, as illustrated in the SEM image (Fig. 2b and Fig. S2b, SI), which will be used as the mobile electrochemical cell to characterize the electrochemical activity of the graphene. The nanoball looked bright because the ions, such as KCl, K_4_[Fe(CN)_6_] and K_3_[Fe(CN)_6_], in the solid electrolyte were dissociative from the water locked inside the crosslinked PAM network during the freeze-drying process. The formed crystals covered the nanoball, resulting in the increased reflection of secondary electrons.

The contact of the nanoball with the ITO surface beneath the nanocapillary is confirmed by the observation of a current increase during the approach process of the nanocapillary to the ITO surface. The hydrogen evolution reaction (HER) was chosen as the model reaction, and 500 mM H_2_SO_4_ was filled in the solid electrolyte inside the capillary. The current traces (Fig. 2c) illustrate a fast current increase upon the contact of the nanoball with the ITO layer. After contact with the surface for 100 ms, the nanocapillary is withdrawn, resulting in a drop in the current, which is close to the phenomenon observed in liquid droplets [30], [31], [32]. To verify the stability of this system, it is easy enough and suitable for most electrochemical surfaces. The relative standard deviation of the current increase during the consecutive 100 contacts is calculated to be 4.21% (Fig. S3, SI), suggesting good reproducibility for the contacts of the nanoballs with the surface.

### 3.2 Resolutions of all-solid SECCM

To characterize the lateral resolution in this solid SECCM, we carried out SECCM scanning on a Au layer with a scanning interval of 50 nm. With an applied positive voltage of 2 V at the Au layer, the Au at the contact region is dissolved through the electrochemical reaction, leaving holes at the surface:

Au - 3e^−^ + 4Cl^−^ → [AuCl_4_]^−^

From the SEM image (Fig. 2d), a regular array of holes is observed with an interval of 50 nm, supporting the electrochemical dissolution of the gold layer during each contact with the nanoball. The diameters of these holes are measured to be 11.8 ± 0.64 nm, which clearly illustrates the spatial resolution of ∼10 nm in the x-y directions using our all-solid SECCM. The relative standard deviation of the size of the holes is calculated to be 5.4%, which ensures accurate lateral resolution in our system.

For the investigation of the vertical resolution (z direction), a Au layer with a thickness of ∼7 nm at the ITO slide is used as the model, as confirmed by atomic force microscopy (AFM, Figure S4,SI). The SECCM image in Fig. 2e and f illustrates a height difference of ∼7.5 nm between the ITO and Au layers, which is consistent with the AFM result. The visualization of this thin Au layer demonstrates a vertical resolution on the order of 7 nm, which could not be achieved by the traditional liquid droplet-based SECCM. Overall, the resolutions in the x-y-z directions are improved down to 10 nm using all-solid SECCM, which offers the feasibility to study the electrochemical activity of 3D materials.

### 3.3 Measuring the electrochemical activity of graphene using all-solid SECCM

After verification of the spatial resolution of our all-solid SECCM, this approach is applied to map the electrochemical activity at the graphene on the ITO slide. A region including ITO, graphene and the wrinkle is selected as the sample surface for scanning. Potassium ferrocyanide/ferricyanide (100 mM) and KCl (500 mM) are contained in the solid electrolyte. The cyclic voltammetry test of this solid electrolyte on the graphene-coated ITO surface shows a pair of typical redox peaks that correspond to the oxidation and reduction of Fe^2+/3+^ (Fig. S5, SI). Although the electrochemical reversibility is reduced in solid electrolytes, the oxidative current from ferrocyanide (at 0.5 V vs. Ag/AgCl) should still be used to study the electron transfer of graphene. Upon contact of the nanoball with the graphene, the current is measured to be ∼500 fA at a potential of 0.5 V. Due to the low current noise of ∼5 fA (Fig. S6, SI), the oxidative current is capable of providing a high-quality image of the electrochemical activity at the graphene surface with a resolution of 10 nm.

**Fig 3:**
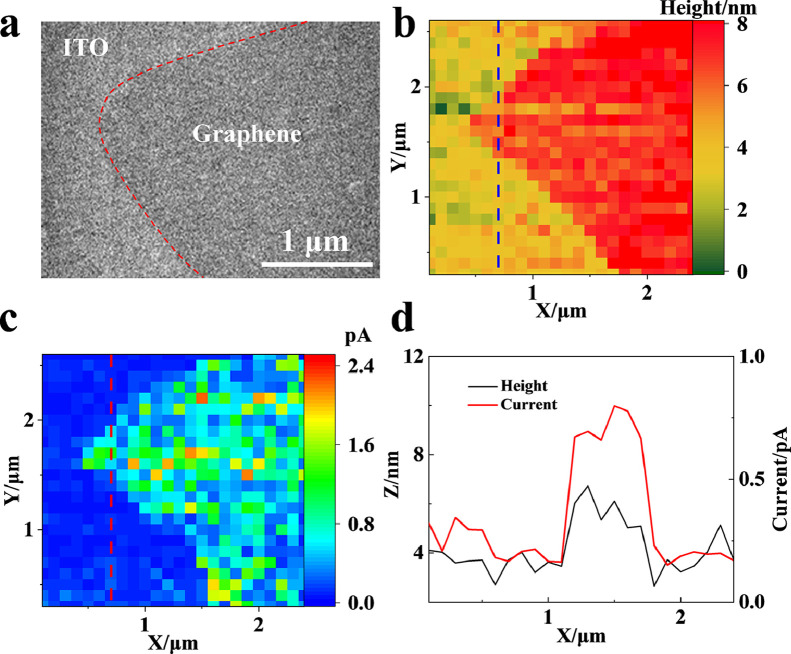
(a) SEM image, (b) SECCM image (morphology), (c) SECCM image (electrochemical activity) of graphene on the ITO slide, and (d) plot of the morphology and current at the red line in Fig. b and c.

SECCM imaging was conducted on microsized graphene on a supporting ITO slide, which was initially observed by SEM (Fig. 3a). Then, the surface was scanned using the nanocapillary with a scanning step interval of 500 nm for fast mapping. By recording the positions of the nanoballs, the morphology of the graphene on ITO is clearly visualized in the SECCM image (Fig. 3b). The thickness of the multilayer edge of graphene is characterized to be ∼5 nm, which is in accordance with the literature results. Additionally, the oxidative currents at each contact region are recorded and illustrated in Fig. 3C. The currents at graphene are significantly higher than those at the ITO surface, exhibiting a faster electron transfer rate at graphene. Importantly, the boundaries between graphene and ITO in the SECCM images of morphology (Fig. 3B) and electrochemical activity (Fig. 3c) coincide (Fig. 3d), which supports the accuracy of the mapping of electrochemical activity at the graphene surface. The realization of the simultaneous mapping of the electrochemical activity and the morphology using all-solid SECCM permits the further study of the electrochemical activity at nanometer-sized wrinkles.

### 3.4 Characterization of the electrochemical activity of the wrinkles on graphene

**Fig. 4:**
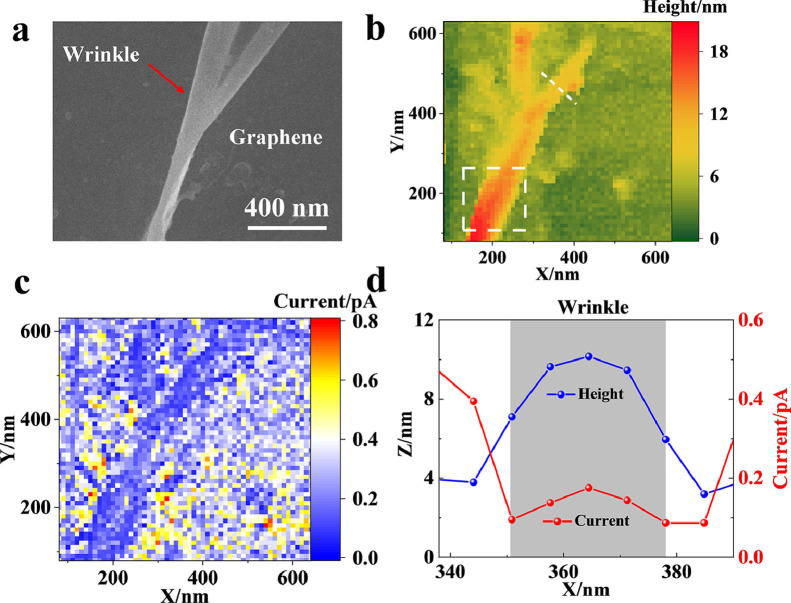
(a) SEM, (b) SECCM (morphology), and (c) SECCM (current) images of the graphene with the wrinkle. (d) Heights and currents of the wrinkle and planar graphene (labeled with the white line in Fig. 4b).

To investigate the electrochemical activity at the wrinkle of graphene, a small area (640 × 640 nm) was chosen for continuous scanning with a step interval of 10 nm. The SEM characterization exhibits a fold with a height of ∼20 nm and a width of ∼80 nm (Fig. 4a), which is the wrinkle of graphene. The SECCM images provide the three-dimensional morphology (Fig. 4b) and currents (Fig. 4c) of the corresponding area, which clearly shows the existence of the wrinkle. The oxidative currents at the interface at the wrinkle are obviously less than those at the flat graphene (Fig. 4d), suggesting a slow electron transfer rate at the wrinkle. Further statistical analysis of the currents of the wrinkle and planar surfaces was determined to be 0.13 ± 0.05 pA and 0.43 ± 0.06 pA, respectively. The successful recording of the currents at different regions of graphene offers local information about the standard rate constants (k_0_) of the wrinkle (0.0034 cm/s) and the graphene surface (0.011 cm/s), which are not easily determined using the traditional electrochemical method.

**Fig. 5:**
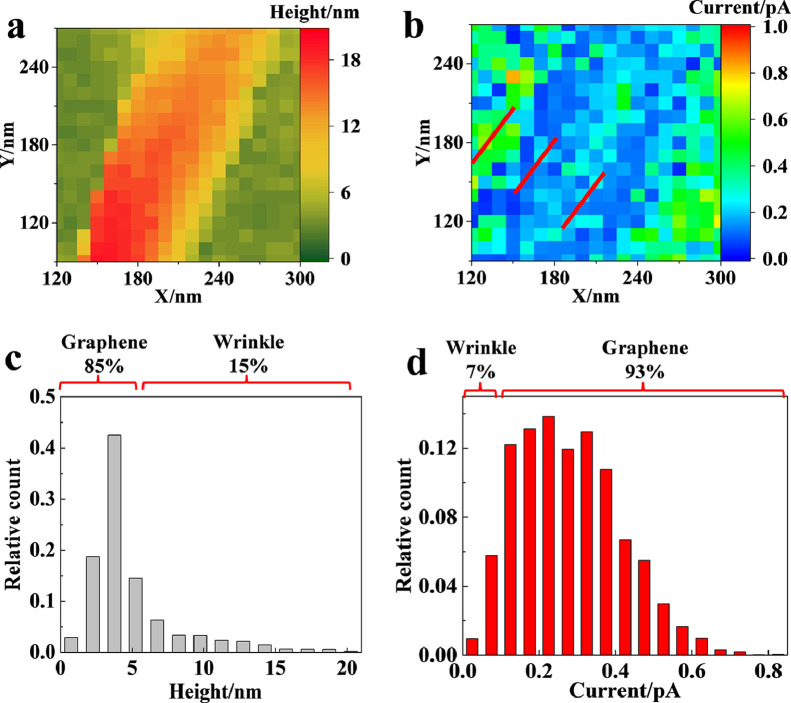
(a, b) SECCM image of the (a) morphology and (b) current of the wrinkle. (c, d) Relative frequency statistics of the (c) morphology and (d) current of the wrinkle.

Importantly, continuous scanning at the wrinkle with a resolution of 10 nm permits fine observation of the currents of the whole wrinkle (Fig. 5a, b). The typical currents at the center and sidewall of the wrinkle and planar graphene (labeled with three white lines in Fig. 5b) are illustrated in Fig. S7 (SI). Compared with the current of 0.10 ± 0.01 pA at the two sidewalls of the wrinkle, the current in this flat center of the fold increases to 0.21 ± 0.02 pA. The observation of different currents illustrates the heterogeneity in the electrochemical behaviors of the wrinkle for the first time. To illustrate more accurate data, relative frequency statistics of the morphology and current are conducted (Fig. 5c and d). Regarding morphology imaging, the regions with heights larger than 5 nm (the wrinkle) account for approximately 15% of the whole scanning area, while the part with low currents (less than 0.1 pA) accounts for only approximately 7%. The difference (∼8% of the wrinkle) between these two percentages should be ascribed to the relatively large current distributed at the center of the wrinkle. Based on this delicate imaging, the standard rate constants of the sidewall and center of the wrinkle are determined to be 0.0026 cm/s and 0.0055 cm/s, which could more realistically reflect the electrochemical feature of the wrinkle.

It is noted that since the center of the winkle with high electrochemical activity is only 30–40 nm in width, continuous scanning without the interval is critical. To illustrate this importance, the wrinkle was scanned with a step interval of 20 nm using the nanocapillary. As shown in Fig. S8, a decrease in only the current of the wrinkle is observed; however, an increase in the current at the center is missing. This result exhibits the advantage of all-solid SECCM that can scan the whole surface without any interval and illustrates the full electrochemical activity of the wrinkle.

Based on the previous results, it is known that wrinkles alter the morphological properties and associated electronic conditions of graphene, hindering electron transport at the surface. In particular, electrons need to climb over the nanoscale wrinkle, which restricts electron transfer [33]. As a result, the charge transfer resistance at the sidewall of the wrinkle (with a small curvature radius) increases, leading to a low electrochemical activity. Moreover, the center of a wrinkle with a large curvature radius is more similar to planar graphene, and thus, the electron transfers and resultant electrochemical activity are expected to be close to the features of the graphene surface. Although the mechanism of electron transfer of the wrinkles has been proposed, direct evidence has not been obtained. Our high spatial SECCM image provides the first and direct evidence to support the proposed mechanism that reveals the heterogeneity of the electrochemical activity of the wrinkles.

## 4 Conclusion

In this paper, all-solid SECCM is established by filling solid electrolytes into nanocapillaries to characterize the morphology and electrochemical activity of the wrinkles on graphene. The lateral and vertical spatial resolutions are down to ∼10 nm, and no scanning interval is present in the image. These new features permit the fine study of the electrochemical activity of nanoscale wrinkles, which is difficult to realize using the current electrochemical imaging methods. Low currents are observed at the two sidewalls of the wrinkles, while relatively high currents are recorded at the center of the wrinkles. The heterogeneous distribution of the currents of the wrinkles reveals the difference in electrochemical activity, which should be important to understand the electron transfer of 3D surfaces with different curvatures. The continuous improvement in temporal resolution is ongoing in the laboratory, which could push the fast investigation of electrochemical materials. Moreover, more electrochemical functions, such as electrochemical impedance spectroscopy (EIS) and cyclic voltammetry (CV), are being adopted in this SECCM platform to improve this type of electrochemical microscopy.

## Declaration of Competing Interests

The authors declare that they have no conflicts of interest in this work.

## Appendix Supplementary materials

Image, application 1
