# Supplementary material for: Highly spatial imaging of electrochemical activity on the wrinkles of graphene using all-solid scanning electrochemical cell microscopy
Source: Fundam Res. 2021 Aug 19;2(2):193–7. doi: 10.1016/j.fmre.2021.08.001 (PMC11197576; doi:10.1016/j.fmre.2021.08.001)
Supplement: Supplementary file 1 [file mmc1.docx]

Supporting Information for

Highly Spatial Imaging of Electrochemical Activity at the Wrinkle of Graphene using All-solid Scanning Electrochemical Cell Microscopy

Rong Jin^1^, Hong-yan Lu^1^, Lei Cheng^2^, Jian Zhuang^2^, Dechen Jiang^1^*, Hong-Yuan Chen^1^

1 State Key Laboratory of Analytical Chemistry for Life Science, School of Chemistry and Chemical Engineering, Nanjing University, Nanjing, Jiangsu, 210023, P. R. China

2 School of Mechanical Engineering, Xi’an Jiaotong University, Xi’an, Shanxi, 710049, P. R. China

Email: [dechenjiang@nju.edu.cn](mailto:dechenjiang@nju.edu.cn)

Figure S1. Cyclic voltammetry of an ITO electrode in liquid state electrolyte and solid state electrolyte with 500 mM KCl. The electrode area is 7 mm^2^. The reference electrode is Ag/AgCl. The scanning rate is 0.2 V/s.

Figure S2. The top SEM view of nano-capillary (A) without and (B) with the solid PAM nanoball.

Figure S3. The currents collected during 100 consecutive contacts with the ITO surface. The capillary is filled with solid state electrolyte containing 500 mM H_2_SO_4_. The voltage applied at ITO surface is -1.5 V.

Figure S4. (A) AFM image of Au/ITO interface; (B) the analysis of the height of Au layer on the white line in Figure S4A.

Figure S5. Cyclic voltammetry of ITO electrode in solid state electrolyte with 500 mM KCl, 100 mM K_3_[Fe(CN)_6_] and 100 mM K_4_Fe(CN)_6_. The electrode area is 7 mm^2^. The reference electrode is Ag/AgCl. The scanning rate is 0.2 V/s.

Figure S6. The open-circuit current of the nano-capillary filled with the solid electrolyte containing 100 mM KCl. The voltage applied at ITO electrode is 0.5 V.

Figure S7. The SECCM currents recorded from the side-wall and the center of the wrinkle, and the planar graphene along three red lines in Figure 5B.

Figure S8. SECCM morphology and current image of graphene/ITO interface tested by solid state electrolyte probe with 500 mM KCl, 100 mM K_3_[Fe(CN)_6_] and 100 mM K_4_Fe(CN)_6_ under 0.5 V. The step between each pixel is 20 nm.
